# Genome-Wide Identification and Expression Analysis of CrRLK1-like Gene Family in Potatoes (*Solanum tuberosum* L.) and Its Role in PAMP-Triggered Immunity

**DOI:** 10.3390/genes16030308

**Published:** 2025-03-04

**Authors:** Yazhou Bao, Ru Zhao, Sixian Hu, Xiaoli Li, Like Wang, Ji Wang, Junbin Ji, Weiduo Wang, Changqing Zhu, Jiajia Chen, Ailing Ben, Jinfeng Peng, Tingli Liu

**Affiliations:** 1 The Nanjing Engineering Research Center for Peanut Genetic Engineering Breeding and Industrialization, School of Food Science, Nanjing Xiaozhuang University, Nanjing 211171, China; yazhou_bao@163.com (Y.B.); zhaoru09@126.com (R.Z.); 2721976618@qq.com (S.H.); lixiaoli@njxzc.edu.cn (X.L.); likewang@njxzc.edu.cn (L.W.); 43826781@qq.com (J.W.); jijunbin@njxzc.edu.cn (J.J.); 26985976@qq.com (W.W.); 2018018@njxzc.edu.cn (C.Z.); benailing@njxzc.edu.cn (A.B.); 2 College of Landscape Architecture, Jiangsu Vocational College of Agriculture and Forestry, Zhenjiang 212400, China; jiajiachen@jsafc.edu.cn

**Keywords:** StCrRLK1L, genome-wide identification, expression analysis, PAMP-triggered immunity

## Abstract

Background: The *Catharanthus roseus* receptor-like kinase 1-like (CrRLK1L) subfamily, a specialized group within receptor-like kinases (RLKs), was initially identified in *C. roseus* cell cultures. CrRLK1L plays an important role in the growth, development and stress response of plants. Although CrRLK1L genes have been characterized across multiple plant species, their biological and genetic functions in potatoes (*Solanum tuberosum*) remains poorly elucidated. Methods: a genome-wide investigation, phylogenetic analysis, chromosome localization, exon–intron structure, conserved motifs, stress-responsive *cis*-elements, tissue-specific expression patterns, and their effects on pathogen associated molecular patterns (PAMPs) induced reactive oxygen species (ROS) production were analyzed. Results: A total of 29 CrRLK1L genes were identified in the *S. tuberosum* genome, unevenly distributed across 10 chromosomes and divided into three groups. Tissue-specific expression analysis revealed seven genes highly expressed in all tissues, while CrRLK1L13 was specific to stamens and flowers. Under stress conditions (mannitol, salt, hormone, and heat), StCrRLK1L genes exhibited diverse expression patterns. Functional characterization in *Nicotiana benthamiana* identified seven ROS suppressors and four ROS enhancers, implicating their roles in PAMP-triggered immunity. Conclusions: This study provides valuable insights into the StCrRLK1L gene family, enhancing our understanding of its functions, particularly in plant innate immunity.

## 1. Introduction

Plant cells constantly respond to a variety of signals from external and endogenous stimuli which are transformed into appropriate cellular and molecular responses, enabling plant cells to adapt to different local environmental conditions [1]. The receptor-like kinase (RLK) superfamily is one of the largest classes of transmembrane receptor protein systems in plant cells, which can receive, sense and process signals [2]. The RLK superfamily is characterized by a ligand-binding extracellular domain with diverse structures and sequences followed by a transmembrane domain and a conserved cytoplasmic serine/threonine kinase domain. Based on the phylogenetic analysis of extracellular regions and domain organizations, RLK divides into several subfamilies, indicating that each subfamily has a different potential function [3].

The *C. roseus* receptor-like kinase 1-like (CrRLK1L) subfamily, a specialized group within receptor-like kinases (RLKs), was first identified in *C. roseus* cell cultures [4]. Compared to other RLKs, CrRLK1Ls are characterized by one or two extracellular malectin-like carbohydrate-binding domains [5,6]. In *A. thaliana*, 17 CrRLK1L members have been characterized, each comprising a signal peptide, an extracellular malectin-like domain, a transmembrane domain and a cytoplasmic kinase domain [5,6,7]. Subsequently, CrRLK1L was also characterized in some crops. For example, 16/17 CrRLK1L members have been characterized in rice [8,9]. In cotton, 44, 40 and 79 CrRLK1L genes have been characterized (*Gossypium raimondii*, *Gossypium arboreum* and *Gossypium hirsutum*, respectively) [9,10]. In soybeans, 38/46 CrRLK1L genes were characterized (*Glycine max*) [9,11].

The CrRLK1L subfamily has been widely studied in *A. thaliana*. The functions of some members of the CrRLK1L family in cell wall integrity coordination, intercellular communication between gametophytes, cell elongation and cell wall sensing have been reported [4]. Among them, FERONIA (FER), THESEUS1 (THE1), HERCULES1 (HERK1) and HERCULES2 (HERK2) were strongly expressed in vegetative tissues and brassinosteroids could also regulate growth and development through them [12]. Previous studies have shown that FER responds to different hormonal signals, including auxin, ethylene, brassinosteroid (BR) and ABA [13,14,15,16]. ANXUR1 (ANX1) and ANXUR2 (ANX2) are the closest FER homologues in *A. thaliana*, involved in the growth and development of pollen tubes [17,18]. In addition, several studies have shown that the CrRLK1L family also plays an indispensable role in plant immunity. For example, ANX1 and ANX2 constitutively associate with FLS2 (flagellin-sensitive 2) and the perception of flagellin epitope flg22 promotes ANX1 association with BAK1 (BRASSINOSTEROID INSENSITIVE 1-associated kinase 1), which interferes with ligand-induced FLS2–BAK1 complex formation to negatively regulate plant immune responses [19]. However, FER positively regulates flg22-mediated responses by facilitating the complex formation between FLS2 and BAK1 [20]. GmLMM1 (lesion mimic mutant 1), a CrRLK1L subfamily gene in soybeans, is involved in pattern-recognition receptor complexes and negatively modulates flg22-induced reactive oxygen species (ROS), one of the early immune responses [21].

The potato (*S. tuberosum* L.), originating in the Andean regions of Peru and Bolivia [22], ranks as the third most vital staple crop globally, following rice and wheat [23]. The susceptibility of potato plants to pathogens during cultivation frequently results in substantial reductions in yield and quality. Notably, late blight, caused by *Phytophthora infestans*, represents one of the most devastating diseases affecting potato production [24]. Currently, the CrRLK1L gene family has been reported to be involved in the regulation of the immune system in a variety of plants, such as FER, ANX1/2 in *A. thaliana* and GmLMM1 in soybeans. However, systematic investigations of the CrRLK1L gene family and its functional association with disease resistance mechanisms remain conspicuously understudied in potatoes. In this study, 29 StCrRLK1L genes in potatoes were identified and analyzed by bioinformatics, including subcellular and chromosome location, phylogenetic relationship with *A. thaliana*, intron–exon structure, conserved protein motif, stress-related *cis*-elements and tissue-specific expression pattern. Finally, we transiently expressed the 25/29 family members in *N. benthamiana* to detect their role in flg22-induced ROS. Our results suggest that seven genes (CrRLK1L6, CrRLK1L8, CrRLK1L9, CrRLK1L11, CrRLK1L15, CrRLK1L16 and CrRLK1L17) strongly suppressed flg22-induced ROS accumulation and four genes (CrRLK1L19, CrRLK1L20, CrRLK1L27 and CrRLK1L28) strongly enhanced flg22-induced ROS accumulation in *N. benthamiana*. This study provides a wealth of information about the StCrRLK1L gene family, and increases our understanding of its functions, especially in plant innate immunity.

## 2. Materials and Methods

### 2.1. Plant Materials and Growth Conditions

*N. benthamiana* were cultured at 23 °C in 70% relative humidity with a 14-h light/10-h dark cycle.

### 2.2. Identification of CrRLK1Ls in S. tuberosum

The *S. tuberosum* proteome was screened using HMMER3.1 [25,26] with two PFAM domains: malectin-like (PF12819) and pkinase-Tyr (PF07714). Full-length protein sequences of 17 *A. thaliana* CrRLK1L genes served as queries for BLASTP [27] searches against the *S. tuberosum* genome annotation. Following redundancy removal, candidate sequences were validated via SMART [28] to confirm domain architecture prior to further analysis.

### 2.3. Sequence Analysis and Chromosomal Localization in S. tuberosum

Gene location and protein length (aa) were calculated using the Phytozome database. Physicochemical parameters (e.g., molecular weight and isoelectric point) of StCrRLK1L proteins were calculated using the ExPASy Compute pI/Mw tool (https://web.expasy.org/compute_pi/ (accessed on 20 December 2024)). Subcellular localization predictions were performed with Plant-mPLoc (http://www.csbio.sjtu.edu.cn/bioinf/plant-multi/ (accessed on 20 December 2024)).

### 2.4. Phylogenetic Analysis and Classification of StCrRLK1Ls

Full-length amino acid sequences of 29 StCrRLK1Ls and 17 *A. thaliana* CrRLK1Ls [5] were aligned using ClustalW (https://www.genome.jp/tools-bin/clustalw (accessed on 20 December 2024)) [29]. Maximum-likelihood phylogenetic trees were generated with MEGA 10.2 [30] with branch support assessed through 1000 bootstrap replicates.

### 2.5. Analysis of Structural Characterization in S. tuberosum

The MEME version 5.3.3 program (https://meme-suite.org/meme/tools/meme (accessed on 25 December 2024)) was used to identify motifs in the StCrRLK1Ls‘ sequence with the following parameters: the maximum number of motifs was set to 6, optimum motif width was set to 30–50, and any number of repeats [31]. The exon–intron structures of the StCrRLK1L genes were obtained from the Phytozome database.

### 2.6. Analysis of Cis-Acting Elements in the Promoter of StCrRLK1L Genes

To analyze the *cis*-acting elements of StCrRLK1L genes, 2-kb promoter regions upstream of the ATG initiation codon for each StCrRLK1L gene were retrieved from the Phytozome database. Putative *cis*-elements were annotated using the PlantCARE online platform (http://bioinformatics.psb.ugent.be/webtools/plantcare/html/ (accessed on 28 December 2024)) [32].

### 2.7. Expression Pattern Analysis of StCrRLK1L Genes

Tissue-specific and stress-response expression patterns of StCrRLK1L genes were analyzed using Illumina RNA-seq data from the PGSC [33]. Tissues included carpels, petals, petioles, stolons, stamens, sepals, tubers, shoots, roots, leaves, immature fruit, mature fruit and mature flowers in DM potatoes. Stress treatments comprised the following: (1) phytohormones: 10 μM IAA, 50 μM ABA, 50 μM GA_3_, or 10 μM BAP for 24 h; and (2) abiotic stresses: 150 mM NaCl (salt), 260 μM mannitol (drought mimic), or 35 °C (heat) for 24 h.

### 2.8. ROS Production Assay

StCrRLK1L genes were PCR-amplified and cloned into the pCAMBIA1300-35S-HA-RBS vector for transient expression in 5- to 6-week-old *N. benthamiana* leaves. After 48 h, leaf discs (8 mm diameter) were excised using a cork borer (Sigma), incubated overnight in 200 μL of water in 96-well plates, and treated with 200 μL of reaction buffer containing 20 μM of L-012 (Wako Chemical), 10 μg/mL of horseradish peroxidase (Sigma), and 1 μM of flg22. Luminescence was quantified using a Tecan F200 microplate reader.

## 3. Results

### 3.1. The Identification of CrRLK1L Subfamily Genes in the S. tuberosum Genome

The Hidden Markov Model (HMM) and BLASTP algorithm search were performed against the annotation of the *S. tuberosum* genome. Finally, 29 CrRLK1L genes were identified in the *S. tuberosum* genome, which were renamed StCrRLK1L1-StCrRLK1L29 based on their chromosomal location (Table 1). The features of the 29 StCrRLK1L gene family members were analyzed, including length of amino acid, protein molecular weight (MW), isoelectric point (pI) and subcellular location (Table 1). The length and physicochemical properties of the StCrRLK1L gene family members varied greatly. The amino acid lengths of the StCrRLK1L genes ranged from 627 aa (CrRLK1L28) to 1188 aa (CrRLK1L4), the molecular weights varied from 69.16 kDa (CrRLK1L28) to 132.56 kDa (CrRLK1L4), and the isoelectric point (pI) values ranged from 5.30 (CrRLK1L25) to 8.40 (CrRLK1L20). Subcellular localization predictions indicated that 29 StCrRLK1L proteins were located in the cell membrane, chloroplast, cytoplasm, mitochondrion and nucleus, suggesting that their functions are diverse. The detailed features of the 29 StCrRLK1L gene family members are listed in Table 1.

### 3.2. StCrRLK1L Gene Distribution on S. tuberosum Chromosome

**Figure 1:**
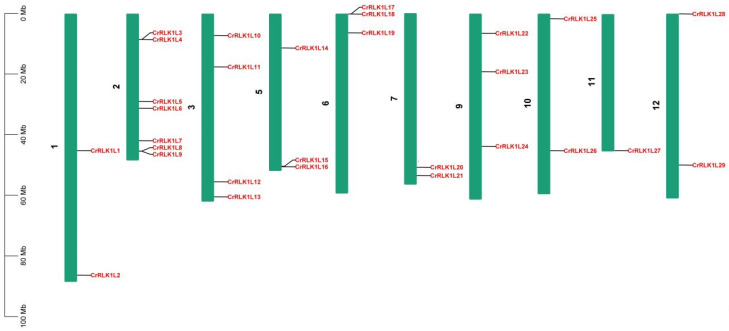
Chromosomal locations of potato StCrRLK1L gene family members. Twenty-nine genes are mapped to 10 chromosomes based on Phytozome annotations.

Detailed information on the location of the 29 StCrRLK1L gene family members used in this study were obtained from the Phytozome (Table 1). Chromosomal mapping revealed that the 29 StCrRLK1L genes are unevenly distributed across 10 of the 12 *S. tuberosum* chromosomes (Figure 1; Table 1). Chromosomes 4 and 8 lacked StCrRLK1L genes, while chromosome 2 harbored the highest density (seven genes). In contrast, only one gene (CrRLK1L27) was found on chromosome 11, which had the least number of StCrRLK1L genes (Figure 1). Four StCrRLK1L members were located on chromosome 3, three StCrRLK1L members were located on chromosomes 5, 6 and 9 and two StCrRLK1L members were located on chromosomes 1, 7, 10 and 12 (Figure 1).

### 3.3. Phylogenetic Analysis of the CrRLK1L Family in A. thaliana and S. tuberosum

**Figure 2:**
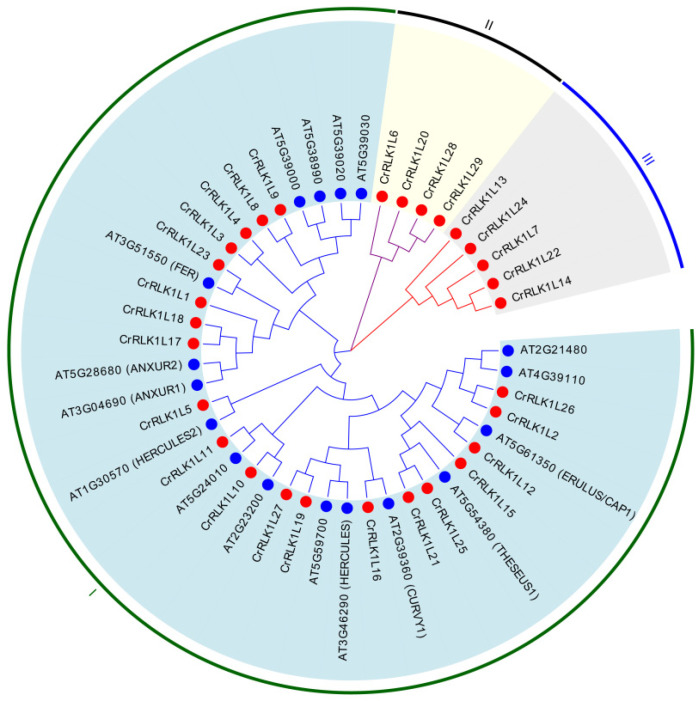
A phylogenetic tree of CrRLK1L-related proteins from *A. thaliana* (blue circles) and *S. tuberosum* (red circles). The tree was constructed using ClustalW alignment and MEGA X with 1000 bootstrap replicates.

To resolve evolutionary relationships, a maximum-likelihood phylogenetic tree was constructed using full-length amino acid sequences of 46 CrRLK1L proteins (29 StCrRLK1Ls and 17 AtCrRLK1Ls). According to the similarity and topology of the amino acid sequences, the 29 StCrRLK1L genes were divided into three main groups named I, II and III. As shown in Figure 2, twenty StCrRLK1Ls and seventeen AtCrRLK1Ls belonged to cluster I, four StCrRLK1Ls belonged to cluster II, and five StCrRLK1Ls were grouped into cluster III.

### 3.4. Gene Structure and Conserved Protein Motif Composition of StCrRLK1L Genes

**Figure 3:**
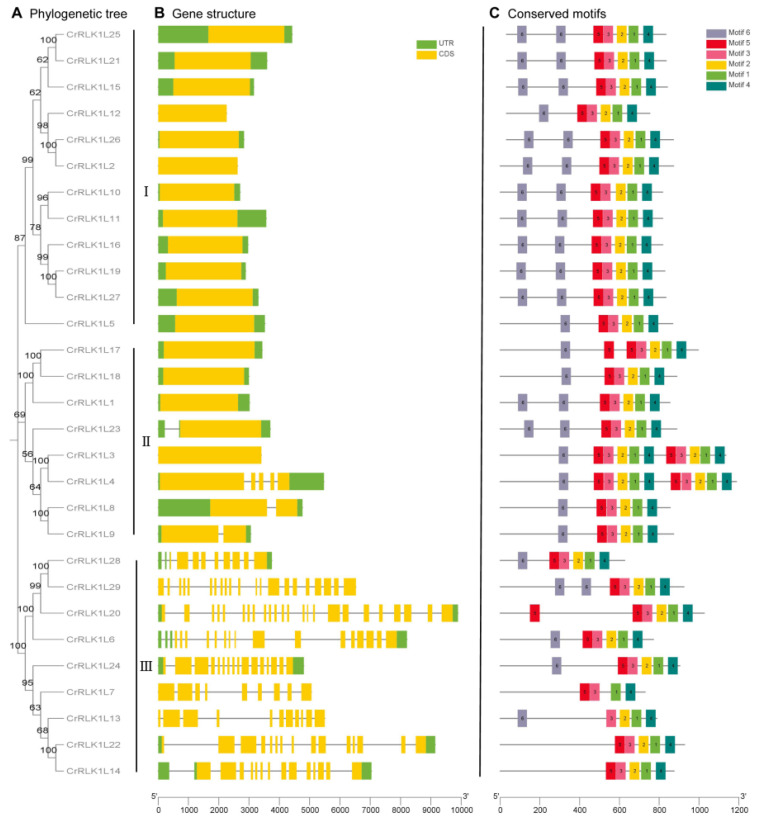
Structural and evolutionary analysis of StCrRLK1Ls. (**A**) Maximum-likelihood phylogenetic tree of 29 StCrRLK1Ls grouped into three subclasses. (**B**) Exon–intron architecture: green (UTRs), yellow (exons), gray line (introns). (**C**) Conserved motif distribution, colored by position in protein sequence.

A phylogenetic tree of StCrRLK1Ls was reconstructed, classifying the 29 genes into three distinct subclasses (I–III; Figure 3A). In order to better understand the evolution of the StCrRLK1L family, the organization of exons–introns was analyzed. Among the StCrRLK1L genes, more than half (16 StCrRLK1L genes, 55.2%) were free introns. Three StCrRLK1L genes (10.3%) had one intron and nine StCrRLK1L genes (31.0%) had two or more introns. Most of the members with introns were distributed in subclass III while all members of subclass I did not contain introns (Figure 3B).

Protein motifs are highly conserved amino acid residues and are considered to have a functional and/or structural role in active proteins. Six conserved motifs were identified in the StCrRLK1L family genes by using the MEME website (Figure 3C). The lengths of the StCrRLK1L-conserved motifs ranged from 48 to 50 aa. The detailed sequences of the six putative motifs are shown in Table S1. All members of the StCrRLK1L genes contained Motif 1, Motif 3 and Motif 4. Motif 2 and Motif 5 were found in 28 StCrRLK1L genes, with the exception of StCrRLK1L7 and StCrRLK1L13. Except for four members (StCrRLK1L7, StCrRLK1L14, StCrRLK1L20 and StCrRLK1L22) of the StCrRLK1L gene family, Motif 6 was found in the other 25 StCrRLK1L genes.

### 3.5. Stress-Related Cis-Elements in the Promoter of StCrRLK1L Genes

**Figure 4:**
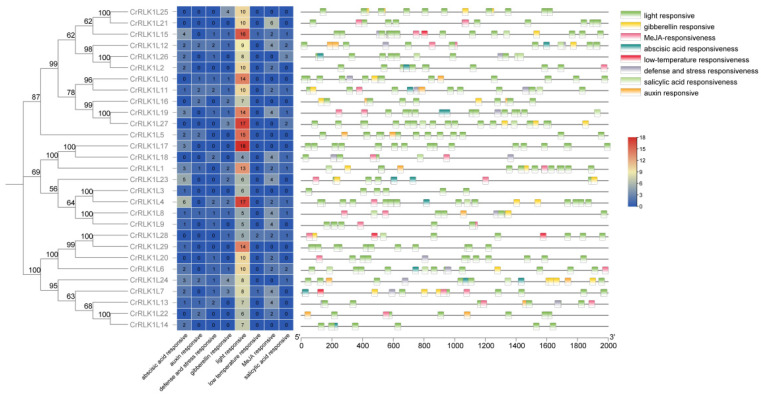
*Cis*-element distribution in the StCrRLK1L promoters. Element positions are scaled relative to the translation start site (ATG).

In order to further analyze the possible regulatory mechanism of StCrRLK1L genes in abiotic and biotic stress response, a 2.0 kb upstream sequence of StCrRLK1L genes was submitted to PlantCARE to detect stress-related *cis*-elements. Eight stress-response elements, including abscisic acid (ABA), auxin, defense/stress, gibberellin, light, low temperature, MeJA and salicylic acid, were identified in the promoter of 29 StCrRLK1L genes (Figure 4).

Our results demonstrate that 29 StCrRLK1L genes have at least seven (CrRLK1L3) *cis*-elements related to stress response, which indicates that the expression of StCrRLK1L genes could be associated with stress responses. Among them, all 29 StCrRLK1L genes contain at least four *cis*-elements related to light response, the most *cis*-elements among all the stress-related elements. Among the twenty-nine StCrRLK1L family genes, only three genes (CrRLK1L7, CrRLK1L15 and CrRLK1L28) have a *cis*-element related to low temperature response, the least among all the stress response elements (Figure 4). The *cis*-elements related to abscisic acid, auxin, gibberellin, MeJA and salicylic acid were found in 20/29, 10/29, 16/29, 18/29 and 13/29 StCrRLK1L genes, respectively (Figure 4). One or more *cis*-elements related to defense and stress response are contained in 15 of the 29 StCrRLK1L family genes (Figure 4). The *cis*-elements analysis shows that 29 StCrRLK1L family genes might participate in different stress responses.

### 3.6. Expression Pattern Analysis of StCrRLK1L Genes in Different Tissues

**Figure 5:**
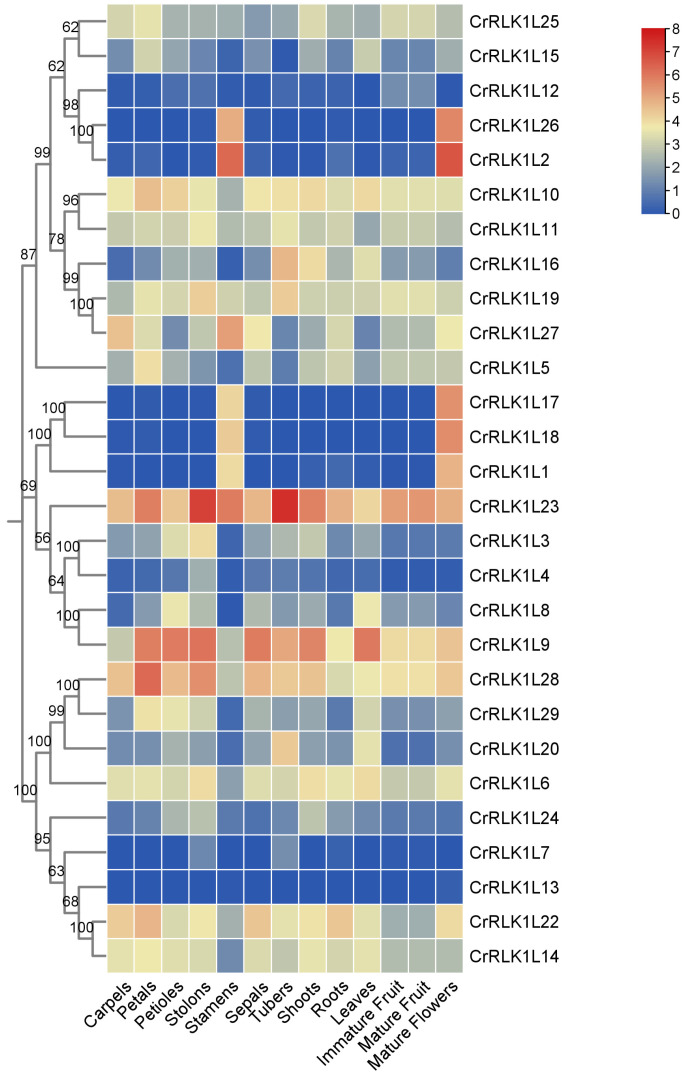
Heat map of StCrRLK1L expression across tissues in DM potatoes. Color intensity reflects log2-transformed FPKM values.

Different genes have different expression levels in different tissues or organs so that they can adjust their physiological processes. To elucidate tissue-specific regulatory patterns, transcript abundances of StCrRLK1L genes were analyzed across 13 organs/tissues: carpels, petals, petioles, stolons, stamens, sepals, tubers, shoots, roots, leaves, immature fruit, mature fruit and mature flowers. As shown in Figure 5, the transcript abundance of 28 of the 29 StCrRLK1L genes were obtained. All of the 28 StCrRLK1L genes were expressed in at least two tissues. The results show that seven StCrRLK1L genes (CrRLK1L9, CrRLK1L10, CrRLK1L11, CrRLK1L19, CrRLK1L22, CrRLK1L23 and CrRLK1L28) were highly expressed in all tissues with FPKM >3. Approximately 20 genes (71.4%) were expressed in all tissues with a different transcript abundance. Strikingly, StCrRLK1L13 exhibited strict tissue specificity, with expression restricted to stamens and mature flowers (Figure 5; Table S2).

### 3.7. Expression Pattern Analysis of StCrRLK1L Genes Under Phytohormonal- and Abiotic- Stress Treatments

**Figure 6:**
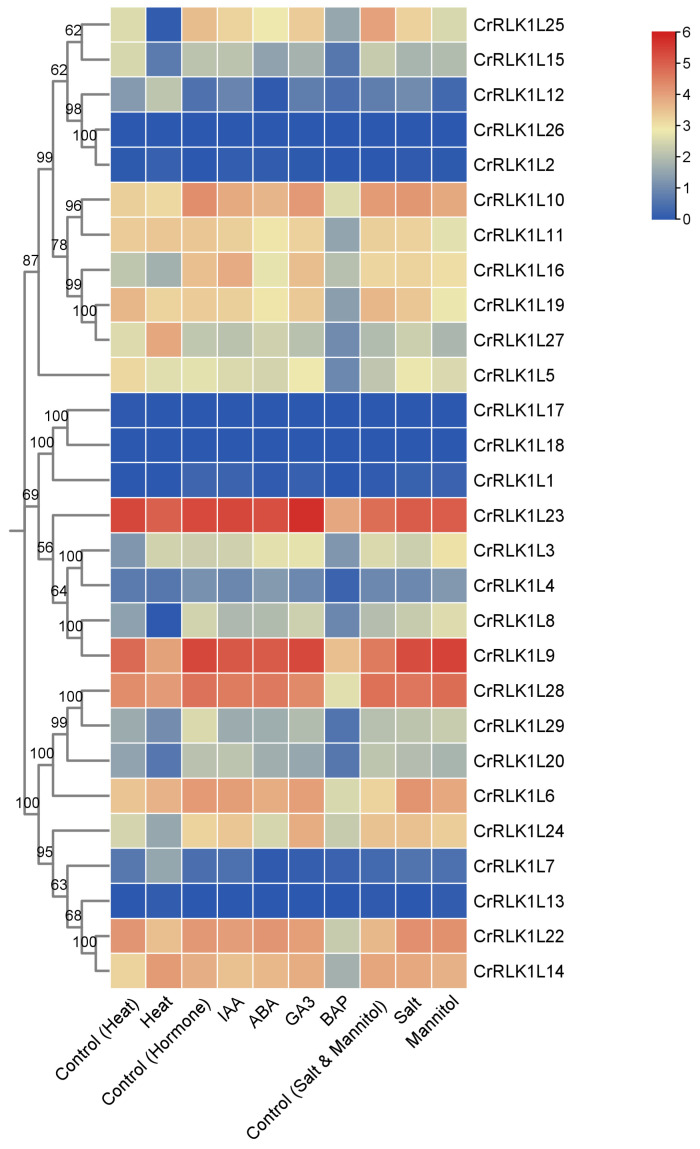
Expression dynamics of the StCrRLK1L genes under stress conditions. The color scale was plotted using the log2 mean of FPKM of each gene.

In order to investigate the role of StCrRLK1L genes in phytohormone and abiotic stress, we analyzed the transcription expression spectra of the StCrRLK1L genes under phytohormonal (10 μM IAA, 50 μM ABA, 50 μM GA_3_ and 10 μM BAP), salt stress (150mM NaCl) and drought induced by 260 μM mannitol and heat (35 °C) treatments. As shown in Figure 6, the transcript abundance of 28 of the 29 StCrRLK1L genes were obtained. The results show that ten (CrRLK1L2, CrRLK1L3, CrRLK1L7, CrRLK1L8, CrRLK1L12, CrRLK1L15, CrRLK1L20, CrRLK1L24, CrRLK1L25 and CrRLK1L27), two (CrRLK1L1 and CrRLK1L6), and three (CrRLK1L1, CrRLK1L12 and CrRLK1L25) StCrRLK1L genes were differentially expressed (|log2(FC)| > 1) under heat, salt, and mannitol treatments, respectively (Figure 6 and Table S3). Among the phytohormonal treatments, one StCrRLK1L gene (CrRLK1L29) was differentially expressed under IAA treatment. Four StCrRLK1L genes (CrRLK1L1, CrRLK1L7, CrRLK1L12 and CrRLK1L29) were differentially expressed under ABA treatment. One StCrRLK1L gene (CrRLK1L7) was differentially expressed under GA_3_ treatment, and twenty-one StCrRLK1L genes were differentially expressed under BAP treatment (Figure 6 and Table S3).

### 3.8. Analysis of the Role of StCrRLK1L Genes in flg22-Induced ROS in N. benthamiana

**Figure 7:**
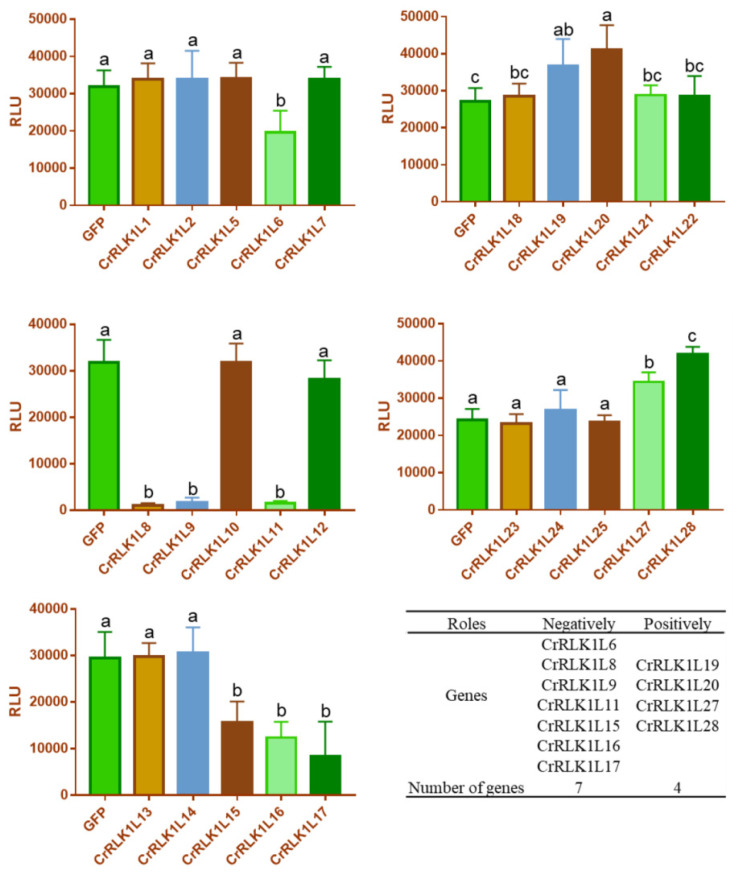
The Modulation of PAMP-triggered ROS by StCrRLK1Ls. The indicated constructs were transiently expressed by agrobacterium-mediated transient expression for 2 days and subjected to flg22-induced ROS examination (mean ± SD, *n* ≥ 8, and one-way ANOVA followed by Tukey’s post hoc test; different letters indicate significant difference at *p* < 0.01). The protein expression is shown in Figure S1.

Several studies have shown that the CrRLK1L family also plays an indispensable role in plant immunity, such as FER and ANX1/2 in *A. thaliana*. FER was reported to interact with FLS2 and positively regulate flg22-induced ROS [20]. AXN1/2 and GmLMM1 negatively modulate flg22-induced ROS [19,21]. In order to further investigate the role of StCrRLK1L genes in plant immunity, 25 of 29 StCrRLK1L family genes were successfully cloned and Western blot analysis showed these proteins were expressed normally in *N. benthamiana* (Figure S1). Next, we investigated the regulatory activity of StCrRLK1L family members in flg22-induced ROS by using a transient expression assay in *N. benthamiana*. We observed that the expression of seven genes (CrRLK1L6, CrRLK1L8, CrRLK1L9, CrRLK1L11, CrRLK1L15, CrRLK1L16 and CrRLK1L17) strongly suppressed flg22-induced ROS accumulation and four genes (CrRLK1L19, CrRLK1L20, CrRLK1L27 and CrRLK1L28) strongly enhanced flg22-induced ROS accumulation in *N. benthamiana* (Figure 7).

## 4. Discussion

CrRLK1L is a subfamily of RLKs found only in plants. They are characterized by one or two carbohydrate-binding malectin-like domains in the extracellular region, a single transmembrane domain and a cytoplasmic kinase domain [34]. These proteins play pivotal roles in plant growth, development and stress adaptation [6]. FER, the prototypical member of the CrRLK1L subfamily, was initially identified as a key regulator of female reproductive development in plants [35]. Structurally or functionally related CrRLK1Ls, including HERK1, HERK2, and THE1, coordinate cell wall integrity maintenance and plasma membrane dynamic homeostasis [36]. ANX1, ANX2, BUDDHA‘s PAPER SEAL1 (BUPS1, AT4G39110) and BUPS2 (AT2G21480) orchestrate the structural integrity of pollen tube cell walls during polarized growth [17,18,37,38]. ERULUS/CAP1, a tip-growth-specific CrRLK1L, is critically required for calcium (Ca^2+^)-dependent modulation of pollen tube elongation and root hair cell wall remodeling processes [39,40]. CURVY1, another CrRLK1L, plays an important role in trichome and tapetal cell morphogenesis, the transition from vegetative to reproductive state and seed production [41]. MEDOS1-4 are associated with regulating plant development in response to the presence of metal ions [42]. In this study, we found that many genes in the StCrRLK1L family are in the same evolutionary cluster with the above genes. In the StCrRLK1L gene family, StCrRLK1L23 (FER-like) was in the same evolutionary cluster with FER; StCrRLK1L19 and StCrRLK1L27 were in the same evolutionary cluster with HERK1; StCrRLK1L5 was in the same evolutionary cluster with HERK2; StCrRLK1L21 and StCrRLK1L25 were in the same evolutionary cluster with THE1; StCrRLK1L1 (ANX1/2-like), StCrRLK1L17 and StCrRLK1L18 were in the same evolutionary cluster with ANX1/2; StCrRLK1L2 and StCrRLK1L26 were in the same evolutionary cluster with BUPS1/2; StCrRLK1L12 was in the same evolutionary cluster with ERULUS/CAP1; and StCrRLK1L16 was located in the same evolutionary cluster as CURVY1, suggesting that these genes may have the same function as their corresponding genes (Figure 2).

Plants must constantly respond to biotic and abiotic stress in order to survive. Detection of biotic or abiotic stress can induce signals from ion fluxes, the production of ROS, the accumulation of Ca^2+^, phosphorylation cascade, and hormonal pathways [43,44,45]. Several studies have shown that the CrRLK1L family plays an indispensable role in plant immunity. While ANX1/2 suppress immunity by disrupting FLS2–BAK1 complexes [19], FER enhances PAMP signaling via FLS2–BAK1 stabilization [20]. StCrRLK1L23 (FER-like) was in the same evolutionary cluster with FER, and StCrRLK1L1 (ANX1/2-like) was in the same evolutionary cluster with ANX1/2, suggesting that these genes may have the same function as FER and ANX1/2 (Figure 2). Abiotic stress adversely affects the growth and development of plants. In response to this pressure, plants respond through physiological, molecular, biochemical and genetic reactions. Many studies show that FER plays different roles in plant stomatal closure, salt stress, mechanical damage and heavy-metal stress [36,46,47,48]. We also observed that the expression of seven genes (CrRLK1L6, CrRLK1L8, CrRLK1L9, CrRLK1L11, CrRLK1L15, CrRLK1L16 and CrRLK1L17) strongly suppressed flg22-induced ROS accumulation and four genes (CrRLK1L19, CrRLK1L20, CrRLK1L27 and CrRLK1L28) strongly enhanced flg22-induced ROS accumulation in *N. benthamiana* (Figure 7). The *cis*-elements analysis shows that twenty-nine StCrRLK1L family genes may participate in different stress responses (Figure 4) and the transcript abundance of twenty-eight of the twenty-nine StCrRLK1L genes showed that ten (CrRLK1L2, CrRLK1L3, CrRLK1L7, CrRLK1L8, CrRLK1L12, CrRLK1L15, CrRLK1L20, CrRLK1L24, CrRLK1L25 and CrRLK1L27), two (CrRLK1L1 and CrRLK1L6) and three (CrRLK1L1, CrRLK1L12 and CrRLK1L25) StCrRLK1L genes are differentially expressed under heat, salt, and mannitol treatments, respectively (Figure 6 and Table S3). These expressions further support StCrRLK1Ls’ role in stress adaptation.

Non-peptide, or classical plant, hormones can affect plant physiological processes at low concentrations and play an important role in regulating plant growth and development. The transportation, accumulation, binding, degradation and inhibition of their signal cascade are regulated at different levels [49,50,51]. FER and ERULUS/CAP1 are closely related to auxin signal response [40,52]. In addition, FER is also negatively regulated by ABA treatment and ethylene synthesis [15,53]. FER, HERK1 and HERK2 are regulated by BR and are important for BR-mediated cell elongation [12]. FER regulates JA and SA immune responses by stabilizing/destroying MYC2, thereby regulating the signal response to JA and SA [54]. The *cis*-elements related to abscisic acid, auxin, gibberellin, MeJA and salicylic acid were found in 20/29, 10/29, 16/29, 18/29 and 13/29 StCrRLK1L genes, respectively (Figure 4). Among the phytohormone treatments, one StCrRLK1L gene (CrRLK1L29) was differentially expressed under IAA treatment. Four StCrRLK1L genes (CrRLK1L1, CrRLK1L7, CrRLK1L12 and CrRLK1L29) were differentially expressed under ABA treatment. One StCrRLK1L gene (CrRLK1L7) was differentially expressed under GA3 treatment and twenty-one StCrRLK1L genes were differentially expressed under BAP treatment (Figure 6 and Table S3).

## 5. Conclusions

In this study, a total of 29 CrRLK1L genes were identified in the *S. tuberosum* genome, renamed CrRLK1L1-CrRLK1L29 based on their chromosomal locations, and were unevenly dispersed on 10 of 12 chromosomes. According to the similarity and topology of their amino acid sequence, the 29 StCrRLK1L genes were divided into three main groups named I, II and III. The StCrRLK1L genes had specific tissue expressions and seven StCrRLK1L genes were highly expressed in all tissues. Approximately 20 genes (71.4%) were expressed in all tissues with different a transcript abundance. Among them, CrRLK1L13 displayed a tissue-specific expression pattern only expressed in stamens and mature flowers. In addition, under the mannitol, salt, hormone and heat stresses, the StCrRLK1L genes displayed different expression patterns. Moreover, the functional characterization of 25 StCrRLK1Ls in *N. benthamiana* identified seven ROS suppressors and four ROS enhancers, implicating these genes in PAMP-triggered immunity. This study provides a wealth of information about the StCrRLK1L gene family and increases our understanding of its functions, especially in innate immunity.

## Figures and Tables

**Table 1:** The characteristics of 29 CrRLK1L gene family members in *S. tuberosum*. pI: theoretical isoelectric point.

Gene Name	Gene ID	Chromosome	Gene Location	Protein Length (aa)	Molecular Weight (kDa)	pI	Subcellular Location
CrRLK1L1	PGSC0003DMG400021842	Chr.1	45253663-45256227	854	95.05	6.39	Nucleus
CrRLK1L2	PGSC0003DMG400043836	Chr.1	86334523-86337144	873	96.03	5.67	Cytoplasm, Nucleus
CrRLK1L3	PGSC0003DMG400041071	Chr.2	8613635-8617042	1135	127.21	7.35	Cell membrane, Cytoplasm, Nucleus
CrRLK1L4	PGSC0003DMG400010577	Chr.2	8619185-8623457	1188	132.56	8.35	Nucleus
CrRLK1L5	PGSC0003DMG400021186	Chr.2	29091367-29093973	868	96.30	5.57	Cytoplasm
CrRLK1L6	PGSC0003DMG400028454	Chr.2	31321077-31328397	772	86.05	6.22	Cell membrane
CrRLK1L7	PGSC0003DMG400030658	Chr.2	41983221-41988281	729	82.42	8.22	Cytoplasm, Nucleus
CrRLK1L8	PGSC0003DMG400001369	Chr.2	45448141-45451011	856	95.43	6.13	Chloroplast, Cytoplasm, Mitochondrion, Nucleus
CrRLK1L9	PGSC0003DMG400001368	Chr.2	45458609-45461396	873	97.31	6.25	Cell membrane, Chloroplast, Cytoplasm, Nucleus
CrRLK1L10	PGSC0003DMG400014875	Chr.3	7287447-7289903	818	91.57	5.80	Nucleus
CrRLK1L11	PGSC0003DMG400026325	Chr.3	17666491-17668947	818	91.27	7.56	Chloroplast, Cytoplasm, Nucleus
CrRLK1L12	PGSC0003DMG400024665	Chr.3	55546289-55548550	753	83.34	6.11	Nucleus
CrRLK1L13	PGSC0003DMG400002602	Chr.3	60521274-60526772	789	87.97	5.94	Cell membrane, Cytoplasm, Nucleus
CrRLK1L14	PGSC0003DMG400010746	Chr.5	11413805-11419243	875	97.96	6.14	Cell membrane
CrRLK1L15	PGSC0003DMG400023419	Chr.5	50431633-50434161	842	92.84	6.59	Chloroplast, Cytoplasm
CrRLK1L16	PGSC0003DMG400023508	Chr.5	50582425-50584881	818	91.31	5.55	Nucleus
CrRLK1L17	PGSC0003DMG400007299	Chr.6	214455-217448	997	108.69	7.10	Nucleus
CrRLK1L18	PGSC0003DMG400007307	Chr.6	219466-222135	889	97.92	5.51	Nucleus
CrRLK1L19	PGSC0003DMG400009689	Chr.6	6421767-6424256	829	90.65	5.77	Nucleus
CrRLK1L20	PGSC0003DMG400017291	Chr.7	50903025-50912626	1027	113.29	8.40	Cell membrane
CrRLK1L21	PGSC0003DMG402019252	Chr.7	53653922-53656429	835	91.64	5.31	Cell membrane, Chloroplast, Cytoplasm, Nucleus
CrRLK1L22	PGSC0003DMG400001732	Chr.9	6573941-6582660	928	102.67	5.76	Cell membrane
CrRLK1L23	PGSC0003DMG400029885	Chr.9	19231605-19234274	889	97.15	5.93	Cell membrane, Cytoplasm, Nucleus
CrRLK1L24	PGSC0003DMG401017656	Chr.9	43843516-43847797	904	101.13	5.78	Cell membrane
CrRLK1L25	PGSC0003DMG400025030	Chr.10	1778266-1780770	834	91.29	5.30	Cytoplasm, Extracell, Nucleus
CrRLK1L26	PGSC0003DMG400003406	Chr.10	45284880-45287498	872	96.70	5.96	Nucleus
CrRLK1L27	PGSC0003DMG400015547	Chr.11	45084629-45087133	834	91.51	5.73	Nucleus
CrRLK1L28	PGSC0003DMG400015391	Chr.12	180926-184123	627	69.16	6.54	Nucleus
CrRLK1L29	PGSC0003DMG400028617	Chr.12	50035429-50041955	924	102.81	6.06	Cell membrane

## Data Availability

The original contributions presented in this study are included in the article/Supplementary Material. Further inquiries can be directed to the corresponding authors.

## Supplementary Materials

The following supporting information can be downloaded at: https://www.mdpi.com/article/10.3390/genes16030308/s1, Table S1. The detailed sequences of the six putative motifs; Table S2. The FPKM value of StCrRLK1L genes in different tissues; Table S3. The FPKM value of StCrRLK1L genes under mannitol, salt, hormone and heat stress in DM potatoes; Figure S1. A total of 25 out of 29 family genes were successfully cloned and Western blot analysis showed that these proteins were expressed normally in *N. benthamiana*.
