# Supplementary figures and images for: Genome-Wide Identification and Expression Analysis of CrRLK1-like Gene Family in Potatoes (Solanum tuberosum L.) and Its Role in PAMP-Triggered Immunity
Source: Genes (Basel). 2025 Mar 4;16(3):308. doi: 10.3390/genes16030308 (PMC11942165; doi:10.3390/genes16030308)

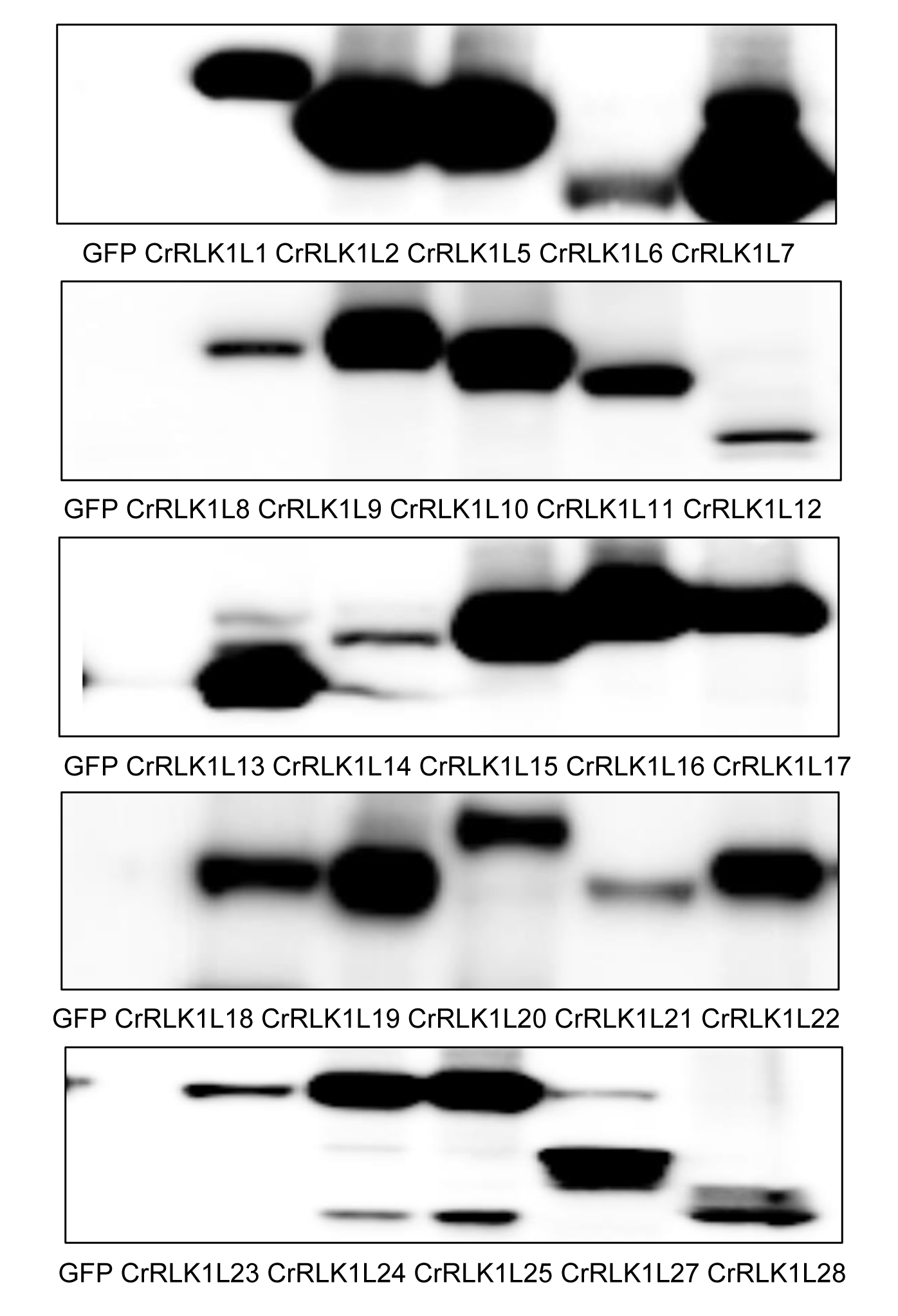

Supplement: Supplementary file 1 [file genes-16-00308-s001.zip › Supplementary Files/Figure S1.tif]
